# Eating Disorder Day Programs: Is There a Best Format?

**DOI:** 10.3390/nu14040879

**Published:** 2022-02-19

**Authors:** Ertimiss Eshkevari, Isabella Ferraro, Andrew McGregor, Tracey Wade

**Affiliations:** 1 Statewide Eating Disorder Service, South Australian Department of Health and Wellbeing, Government of South Australia, Adelaide, SA 5000, Australia; ertimiss.eshkevari@sa.gov.au (E.E.); isabella.ferraro@sa.gov.au (I.F.); andrew.mcgregor2@sa.gov.au (A.M.); 2 Blackbird Initiative, Órama Research Institute, Flinders University, Adelaide, SA 5042, Australia

**Keywords:** eating disorder, anorexia nervosa, bulimia nervosa, other specified feeding and eating disorder, day program, day treatment, stepped care, autonomy

## Abstract

The use of a Day Program (DP) format (i.e., intensive daily treatment with no overnight admission) has been shown to be an effective treatment for eating disorders (EDs). The disadvantages, however, include higher cost than outpatient treatment (including costs of meals and staff), greater disruption to patients’ lives, and the use of a highly structured and strict schedule that may interrupt the development of patients’ autonomy in taking responsibility for their recovery. This study investigated whether reducing costs of a DP and the disruption to patients’ lives, and increasing opportunity to develop autonomy, impacted clinical outcomes. Three sequential DP formats were compared in the current study: Format 1 was the most expensive (provision of supported dinners three times/week and extended staff hours); Format 2 included only one dinner/week and provision of a take-home meal. Both formats gave greater support to patients who were not progressing well (i.e., extended admission and extensive support from staff when experiencing feelings of suicidality or self-harm). Format 3 did not provide this greater support but established pre-determined admission lengths and required the patient to step out of the program temporarily when feeling suicidal. Fifty-six patients were included in the analyses: 45% were underweight (body mass index (BMI) < 18.5), 96.4% were female, 63% were given a primary diagnosis of anorexia nervosa (or atypical anorexia nervosa), and mean age was 25.57 years. Clinical outcomes were assessed using self-reported measures of disordered eating, psychosocial impairment, and negative mood, but BMI was recorded by staff. Over admission, 4- and 8-week post-admission, and discharge there were no significant differences between any of the clinical outcomes across the three formats. We can tentatively conclude that decreasing costs and increasing the opportunities for autonomy did not negatively impact patient outcomes, but future research should seek to replicate these results in other and larger populations that allow conclusions to be drawn for different eating disorder diagnostic groups.

## 1. Introduction

A day program (DP) for eating disorders (EDs), also known as partial hospitalisation [1], is considered effective in terms of fostering remission. Eating disorders typically treated in DP settings include anorexia nervosa, where patients are underweight and may or may not exhibit other disordered eating behaviours such as purging or binge eating, and bulimia nervosa, non-underweight patients where binge eating occurs in conjunction with behaviours designed to offset weight gain, such as purging, exercise or fasting. Lifetime prevalence of these two disorders in females has been estimated at 3.0% to 4.6% and 4% to 6.7% [2].

DPs were first introduced as a new service model for eating disorders in 1989 [3] and described as intensive outpatient care for patients previously considered to require inpatient treatment. The first DP to be evaluated was described as a 2- to 4-month program, 5 days a week, with 10 to 12 patients at any one time. Nutritional stabilisation is considered an essential component of DP, with calories typically divided between three meals and 2–3 snacks per day, adjusted to suit the individual’s age, height, and need for weight gain. Psychological treatment is also considered essential, offered via group but not individual therapy, and typically each day is highly structured, alternating eating together as a group with group therapy. The clear advantage of such an approach is that it is more cost-effective than inpatient treatment.

Since this initial description, many DPs have been established around the world and while adopting a similar approach to that originally described, also report variations in the program such as number of days, number of meals, and addition of individual sessions [4] or use of mandated weight gain required to remain in a program versus recommended weight gain [5]. Published evaluations of changes over admission to discharge show significant increases in weight (for those who are underweight) and significant improvements in psychological functioning. Reporting of weight gain varies greatly between studies, but generally BMI increases on average by 1 to 2 kgs/m^2^, with mean increases of 5 or more kilograms [4,5,6]. Subsequent reports have divided weight change into slow and rapid response, with the latter defined as those patients who gain more than 4 kg in the first 4-week period of DP [4].

While randomised controlled trials are sparse, three exist showing support for the DP format. A comparison of inpatient and DP treatment for adolescents with anorexia nervosa suggested equivalent outcomes but with better outcomes for the DP with respect to mental wellbeing and psychosexual development [7]. An examination of adults with bulimia nervosa that had not responded to 2 years of outpatient therapy again showed equivalent outcomes between inpatient treatment and DP, with remission favouring DP at 41% compared to 33% in the inpatient group [8]. A comparison of DP and outpatient treatment for adults with various eating disorders showed better outcomes for diagnostic behaviours in the DP group [9]. Three further comparisons (non-randomised) of DP to outpatient therapy show equivalent outcomes [10,11,12] except for better outcomes for DP with respect to depression [10], and binge episodes after treatment [12].

A recent critique of DP [13] suggested that there are potential disadvantages to consider. These include costs (patient time, staffing, food for meals), and undermining patient autonomy through use of a structured and strict schedule of programs and menus. Other issues that have been identified include isolation during admissions from family, friends, and support networks, which results in increased reliance on the company of fellow patients [14], which may serve to maintain the illness by increasing identification with the ED and its valued nature [15]—referred to hereafter as social isolation. The purpose of the current study was to investigate whether adjustments to DP aimed at reducing these disadvantages impacted the outcomes of the program. These adjustments were sequential, and included: (1) decreasing costs related to staff and meals, and (2) increasing opportunity for patient autonomy for recovery through use of pre-determined admission lengths, disallowing patients to socialise with other patients outside of DP, and limiting crisis support (support from staff when experiencing feelings of suicidality or self-harm). The outcomes measured included disordered eating, impairment caused by the ED, negative mood (depression, anxiety, and stress), and BMI.

## 2. Materials and Methods

### 2.1. Design

**Figure 1:**
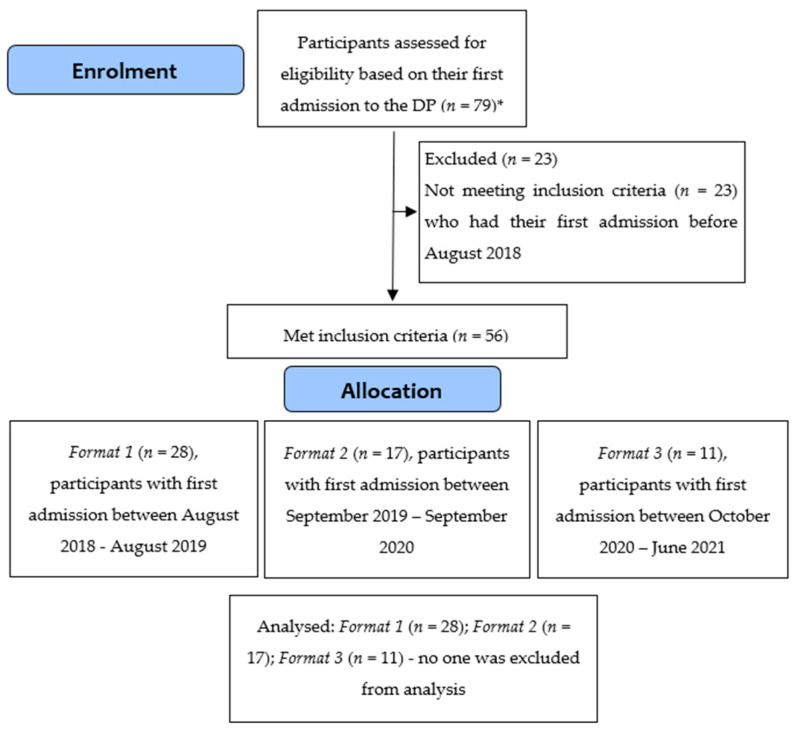
CONSORT diagram of participant flow. * Exception of *n* = 1 participant who had their second admission in 2018 included in the analyses in lieu of their first admission in 2017.

The study design is a sequential case series which compared three different formats of a DP (first established in 2014) over time (shown in Figure 1). The three DP formats (detailed in the Interventions section below) ran sequentially. Only first admissions were included in the analyses, aside from one participant who was found after completion of this study to have been previously admitted to DP; in this case, data from their second admission were collected and used. All participants only appeared once in the dataset, thus ensuring no overlap of participants in the analyses of the three different formats as any previous and/or subsequent admissions were excluded. Change was compared across the three formats over four time points: admission, 4- and 8-week post-admission, and discharge. Given the average duration of admission was 9 to 12 weeks, the timing of discharge was likely to be less than the 4-week intervals between the other data points.

### 2.2. Interventions

All three formats included the following components: The program was structured as group-based treatment with the following eligibility criteria: (1) medically stable individuals (normalization of vital signs, cardiac stability, resolution of electrolyte abnormalities, ability to complete activities of daily living that allows for transition to a lower level of care), (2) a BMI at or above 15, (3) aged 15 years and above, (4) an established primary ED diagnosis of anorexia nervosa, bulimia nervosa, or other specified feeding or eating disorders (OSFED). Diagnoses of avoidant/restrictive food intake disorder and binge eating disorder were excluded. The program ran over 4 days/week with a maximum of 8 patients as rolling admissions. Patients were admitted by their place on the waitlist only, and not according to diagnosis or other aspects of clinical presentation. Meal support and supervision were provided for all meals and snacks eaten on the premises (see Table 1 or an example of a weight restoration meal plan). This entailed providing a supportive environment to achieve participants’ nutritional goals at mealtimes and using skills of role modelling (staff eating with patients), boundary setting, communication and coaching.

Each day in the DP was structured around therapeutic groups, including psychological groups grounded within evidence-based treatment for eating disorders, dietetic groups, as well as other groups (see Table 2 for description of groups). Patients additionally received a weekly one-on-one session with a clinician to discuss individual issues.

They were required to see their general practitioner regularly in order to monitor physical health, given the serious medical sequalae of being underweight. For care continuity, patients were required to maintain regular contact with an external clinician (e.g., psychologist, psychiatrist, mental health care coordinator) during their admission.

A “flag policy” system was utilized, which included abiding by mandated aspects of the DP to remain in the program: for example, weight gain if underweight, genuine attempts at completing all meals and snacks in DP, and consistent progress in meeting treatment goals. Once three “flags” were awarded, the patient’s admission in DP was suspended for two weeks in order to review their ability and motivation to engage in DP treatment and decide whether to resume their admission.

In the standard format (Format 1, from August 2018 to August 2019), admission lengths were determined clinically on an individual case-by-case basis, with a reduction in intensity over the last weeks of the admission to manage the transition to a lower level of care at discharge. Additional meal support for morning tea was also provided in the early weeks of admission to help establish normalization of regular and adequate eating. Supported dinners were provided on three evenings and the program operated for 25.25 h per week. Admission lengths were generally based on purpose of admission such as weight status (e.g., if need to restore weight, may base admission length on duration to achieve this as well as some additional time to experience weight stabilisation). Factors that would increase or extend admission length would include demonstrating good progress with treatment goals and an extension in admission in order to work specifically towards achieving remaining/identified goals. Factors that would reduce admission length would include lack of progress and consistent difficulties in meeting treatment goals. The main aspects of the program have been described and evaluated previously [16].

The second format (Format 2, from September 2019 to September 2020) reduced the provision of supported dinners from three times per week to once a week and provided one take-home dinner per week to promote and generalise the practice of eating adequately. This reduced the costs associated with catering, overtime staffing for evening meals, as well as reduced program time by 3 h to 22.25 h per week. Fewer supervised meals could, however, reduce the weight gain achieved by the DP.

The third format (Format 3, from October 2020 to July 2021) additionally aimed to support the development of patients’ autonomy in taking responsibility for recovery by dismantling any program aspects that rewarded slow progression in treatment; namely: (1) establishment of pre-determined admission lengths prior to commencement of DP, particularly for those in the healthy weight range or who had previous admission(s), which consequently served to increase service capacity; (2) the addition of disallowing patients to socialise with other patients outside of DP to the list of mandated program aspects, as this seemed to foster social isolation and identification with the illness; and (3) limiting provision of crisis support related to self-harm and suicidality, requiring patients to temporarily cease DP until they were able to re-engage in treatment. The time period for this format was 3 months shorter than DP Format 1 and 2. An overview of adjustments to the DP are outlined in Table 3 below.

### 2.3. Participants

Figure 1, a CONSORT diagram, depicts the flow of 56 participants across three formats: 28 (50%) in Format 1, 17 (30%) in *Format 2*, and 11 (20%) in Format 3. Across the formats, 25 (45%) participants commenced DP while underweight (BMI < 18.5), and the mean admission BMI of the remaining participants was 22.20 (SD = 3.24). All but 2 were female (96.4%), and 35 were given a primary ED diagnosis of anorexia nervosa (or atypical anorexia nervosa), 4 of bulimia nervosa, and 17 of OSFED. Mean age of participants was 25.57 (SD = 8.89) years and the mean duration of ED was 7.90 (SD = 8.22) years (latter based on *n* = 46).

### 2.4. Repeated Measures

#### 2.4.1. Eating Disorder Examination Questionnaire

The Eating Disorder Examination Questionnaire (EDE-Q 6.0) [17] was used to assess global eating disorder symptomology. Questions survey the last 28 days and are measured on a seven-point Likert scale with scores ranging from 0 to 6, where a higher score indicates either a greater frequency or severity. The EDE-Q provides four subscales—restraint, eating concern, shape concern and weight concern—and together these provide a global score. The EDE-Q has been validated in clinical ED populations and the general population without ED symptoms and has demonstrated sound psychometric properties and ability to discriminate between ED cases and non-cases [18]. In the present study, Cronbach’s α of the global EDE-Q subscale was 0.93.

#### 2.4.2. Clinical Impairment Assessment

Psychosocial impairment caused by the ED symptomology was assessed using the 22-item Clinical Impairment Assessment (CIA) [19]. This self-report measure surveys the last 28 days, with items covering impairment across mood and self-perception, cognitive functioning, interpersonal functioning, and work performance. Each item is rated on a four-point Likert scale from “not at all” (0) to “a lot” (3). The CIA provides a single index of the severity of psychosocial impairment with scores ranging from 0 to 48; higher scores are indicative of greater psychosocial impairment. A CIA global impairment score of 16 is used as a cut-off point for predicting ED status [14]. The CIA has been previously validated in a population of young women referred to an ED service and demonstrated acceptable psychometric properties [20]. Cronbach’s α for the present study was 0.92.

#### 2.4.3. Depression Anxiety and Stress Scales

The Depression Anxiety and Stress Scales (DASS-21) [21] is a 21-item measure with items rated on a four-point Likert scale ranging from “did not apply to me at all” (0) to “nearly every day” (3), with higher scores reflecting greater negative mood. Use of the three subscales (depression, anxiety, and stress) have been validated [22] and Cronbach’s α in the present study was 0.92.

#### 2.4.4. Body Mass Index

Participants’ weight and height were used to calculate BMI (kg/m^2^). These values were taken from clinicians’ notes at each assessment time point, where participants were weighed in session. Participants were weighed once each week during a Physical Recovery group. They were seen individually by a clinician to carry out collaborative weighing, review of their meal plan adherence, target behaviours and risk.

### 2.5. Baseline Measures

To assess motivation, three visual analogue scales were administered whereby participants were asked to mark along a horizontal line ranging from 0 “not at all” to 100 “very much” how (1) motivated they were to recover, (2) ready they felt to change their eating and weight, and (3) confident they felt that they would succeed if they decided to change. This was scored out of 100. Higher levels of motivation have predicted better treatment outcome in EDs [23], and if this were different between the patients across the three formats, it would provide a confounding reason for any differences between the formats.

### 2.6. Statistical Analyses

Linear Mixed Model (LMM) analyses were performed using IBM SPSS, Version 27 to evaluate within group changes between formats over the course of treatment. This type of analysis retains all participants in a condition, even if they are missing data across different time points. Time and format and the interaction between these two terms were entered as fixed effects. Change over time in each format was examined for our three self-report variables for all participants (disordered eating, impairment, and negative mood), whereas BMI was only examined for those who started program with a BMI < 18.5 as this was the only group where all participants needed to increase weight. Within-group effect sizes were calculated from the start of treatment to discharge, using a procedure recommended by Morris [24] that involves calculating an effect size for single-group pre–post designs by accounting for the within-group correlation between pre- and post-test scores. Small effect sizes were between 0.30 and 0.50, medium effect sizes were between 0.50 and 0.80, and large effect sizes were >0.80, as per custom [25].

## 3. Results

### 3.1. Baseline Characteristics

The three non-overlapping groups are described and compared in terms of demographics in Table 4, showing no significant differences across the people in the three formats at commencement of the day program. This allows us to rule out competing reasons for any differences observed across the different formats.

### 3.2. Change over Time in Each Format

**Figure 2:**
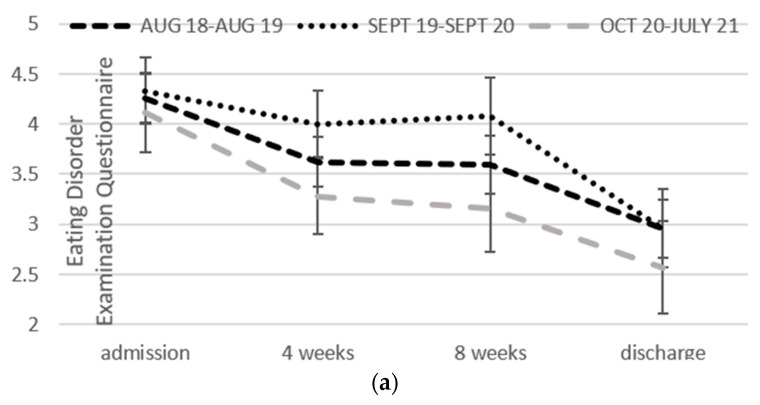

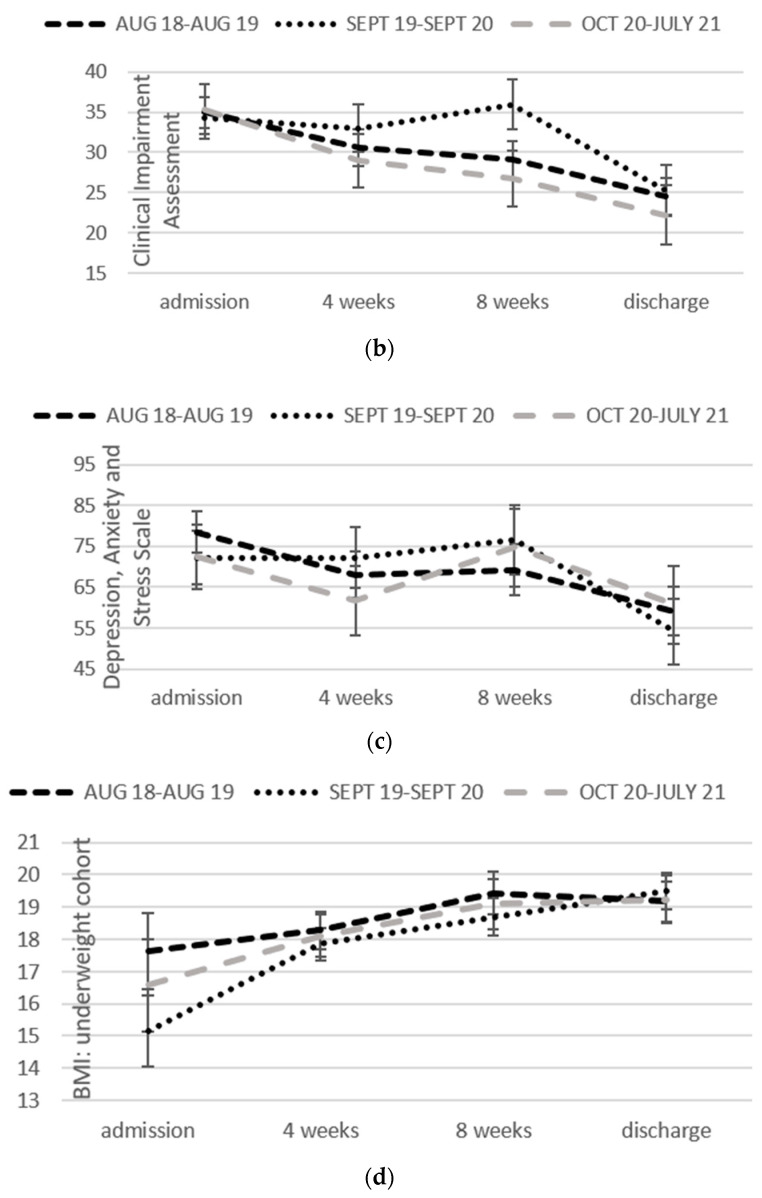
Change over the program for each of the three time periods, August 2018 to August 2019, September 2019 to September 2020, October 2020 to July 2021 in: (**a**) Global levels of disordered eating. (**b**) Impairment due to the eating disorder. (**c**) Depression, anxiety and stress. (**d**) BMI in underweight people (*n* = 24).

A main effect of time was noted for all variables, indicating significant improvements from admission to discharge, which were not associated with any interactions (i.e., a group changing at a different pace from another over time), shown in Figure 2a–c for all participants and Figure 2d for underweight participants only. Given there was no difference between formats, we examined the effect size associated with improvement from admission to discharge across the whole groups, summarised in Table 2. In each case, the 95% confidence intervals for the effect size do not cross zero, which indicates significant improvement.

In Table 5, we also report weight (kilograms), showing an average increase of 6 kg in the underweight cohort, which is commensurate with other DPs [6]. This is associated with a very large and significant effect size.

## 4. Discussion

This research was a pragmatic evaluation, i.e., evaluation of the effectiveness of an intervention in the real world setting where the intervention was adjusted in response to evidence, clinical expertise, and patient preference. Sequential adjustments to the DP format included: (1) reducing costs associated with service provision (staffing and meals) and (2) improving the opportunity to develop autonomy in the recovery process through providing pre-determined admission lengths, prohibiting socialisation with other patients outside of program, and limiting crisis support. Both adjustments transferred greater responsibility to the patient for change. While we did not make directional hypotheses, previous evaluations of ED treatment which included DPs [26] showed that greater support for patient autonomy increased the motivation of patients, which leads to better outcomes. Extrapolation from the critique offered by Ali and colleagues [13] would also suggest that such changes might enhance outcomes.

Results demonstrated there were no significant differences in clinical outcomes when comparing the three DP formats. Overall, the effect sizes achieved over time, regardless of the format patients experienced, showed large decreases in disordered eating and the impairment associated with disordered eating, a large increase in BMI in the underweight patients, and a moderately improved mood. Given there is no interaction between time and format, results indicate that a reduction in costs associated with the program (i.e., reducing intensity and meal support) does not negatively impact patient outcomes. Importantly, this includes weight gain, which intuitively we might think would be smaller when less meals are offered on site. Moreover, such changes provided benefits to the functionality of the service, including reducing costs and staff overtime by reducing program hours and dinner meal supports. Reducing staff burden of working evening/atypical hours may consequently improve wellbeing and may also decrease likelihood of burnout [27,28].

Results also demonstrated that additional approaches to increasing the responsibility and autonomy of the patients (i.e., pre-determined admission lengths that were not lengthened when weight gain was not adequate, requiring patients not to socialise with other patients outside of DP, limiting provision of crisis support) did not adversely impact outcome. Given the trends noticed with respect to two of our outcomes—disordered eating and clinical impairment—investigation of a larger sample may even indicate some superiority of providing greater autonomy.

Overall, in the context of previous DP evaluations [4,5,6,26], we note that outcomes achieved across the three formats are commensurate with those previously reported, suggesting DPs are reliable treatment options for patients with an ED. It is also important to note that there does not appear to be any change in patient profile over time in terms of patient characteristics and psychopathology as there were no significant differences between participants in the three DP formats. Thus, outcomes can be more confidently attributed to the treatment formats provided. In the future, it would be valuable to compare the outcomes of the DP in the current study to inpatient and outpatient treatments, as it has been consistently identified in the literature that there is limited research on clinical outcomes and, in particular, comparisons of different intensities of treatment [13].

There are limitations in the reported research, which should be considered when interpreting the results. First, we have small numbers and, therefore, limited power to detect significant interactions. Larger studies are required to more definitively assess the impact of increasing patient responsibility in DP settings. This limited power is particularly true for our BMI variable, given that less than half our sample started the DP underweight. This issue also affects the power to look at different diagnostic groups, or to adjust for baseline variables such as BMI when examining change. It is possible that different diagnostic groups may respond differently to the changes introduced in the DP format. However, such an evaluation would require such a greater number of participants that is difficult to obtain from one DP. A second limitation is that there are no follow-up data, and future research needs to examine the impact of program format on longer-term maintenance of gains acquired in the DP setting. Third, the passage of time may be confounded with improving clinician skill, which may cancel out any detrimental impact of program change, although the program is also staffed by placement students and new clinicians. We also note that COVID emerged over the term of the second format, and so effects of time may also be confounded with the impact of COVID. Fourth, the nature of the clinical service setting and the uncontrolled nature of this investigation constitute another limitation. For example, the clinical decision-making process in DP *Format 3* of pre-determining DP admission lengths was not systematic and based on factors such as weight status, previous admissions and treatment history. It was also not provided for each participant. Therefore, it would be valuable for future research to be systematic with clear criteria in order to interpret outcomes more clearly. Additionally, due to the rolling nature of the program, a number of patients would not have completed their admission prior to the revised format starting. Thus, there is some overlap across formats for a small number of patients between Format 1 and *2* (*n* = 4) and Format 2 and *3* (*n* = 1). It is also not possible to assess whether Format 3 had any differential impact on risk management and therapy-interfering factors, as it was intended to do. While we did not measure this specifically to quantitatively compare and evaluate outcomes, the clinical impression is that by including these refinements to DP at the outset, it means that participants who had escalations in risk (defined as increased urges related to suicidality or self-harm) were stepped out of the program until they could adequately manage this risk without the need to attend an emergency service setting. This seemed to motivate patients to utilise effective coping skills in order to remain in DP. Secondary gains of additional individual support from clinicians and time out from DP groups for such support were not enabled or accommodated. It also mitigated staff burnout related to providing additional and unscheduled time with patients to manage risk. Finally, while the changes made to DP were designed to reduce treatment and service costs, the financial cost reduction could not be quantified in this study. While these limitations impact the interpreting of results, this process of service evaluation is essential in investigating clinical outcomes and for service development purposes. While being a pragmatic evaluation, the current research addresses an important gap in the field of evaluating DP formats.

## 5. Conclusions

In conclusion, this research has demonstrated that reducing associated costs of DP and increasing opportunity for the development of participant autonomy does not negatively impact on patient outcomes. Instead, with further evaluation, it may demonstrate better outcomes with respect to eating disorder and general psychopathology. Additionally, it provides important benefits to the service in terms of reducing associated costs and staff burnout. While many case series evaluations of DPs exist [1], there has been little attention paid to empirically understanding which format and components of DP can produce the best outcomes [4,25], and further research that can identify the best formats for specific groups of patients is required.

## Figures and Tables

**Table 1:** Day program meal plan for weight restoration.

Meal	Content
Breakfast	1 fruit: ½ cup of juice/½ cup tinned/1 large piece of fresh/6 prunes/1 tbsp dried fruit
	plus cereal: 1 cup of flakes/2 weetbix/1 sachet porridge/ 1/3 cup muesli; with 1 cup of milk
	plus 2 pieces of toast both with 1 tsp butter and 1 tsp spread—jam/honey
Morning Tea	Snack item(s) between 250 and 350 calories
Lunch	Sandwich made with 2 slices of bread with spread—either margarine or mayonnaise plus 2 protein fillings (50 g meat, small can of fish in oil, 1 hard-boiled egg, 1 tub Hommous, 50 g Tofu, 1 slice full fat cheese) Plus salad, 2 or more varieties, enough of each to cover a slice of bread.
	Dessert: Dairy choice from your Snack List
	Drink: Any fruit juices/drink (200–250 mL) or Flavoured milk (200–250 mL)/Soy/flavoured (250 mL)/Soft Drink (250–375 mL)
Afternoon Tea	Snack item(s) between 250 and 350 calories
Dinner	A balanced hot meal will include
Guidelines for appearance of meal	¼ dinner plate protein
	¼ dinner plate carbohydrate
	½ dinner plate vegetables/salad (dressed)
Guidelines for serves of protein	1 red meat steak (palm size in length and thickness), 2 lamb loin chops, 1 med pork chop, 3 slices roast meat, ¼ of a chicken, 1 small chicken breast.
	1 × ’hand’ size piece of fish (150–200 g)
	1 cup (when cooked) chickpeas/lentils/soybeans/baked beans
	2 vegetable/lentil burgers/patties
Guidelines for serves of carbohydrate foods	Tofu 150 g; Quorn 150g mince/2 patties/3 sausages/1 cutlet
Dessert	1 cup of cooked rice /pasta/quinoa/couscous/noodles/mashed potato
	1 medium potato cooked in any way, 2 slices of bread or 1 crusty roll
	Dessert as per current snack list
Supper	Snack item(s) between 250 and 350 calories

**Table 2:** Overview of group content.

Group Name	Description
Review	First group of each day. Review of meal plan adherence and ED behaviours since last in DP, problem-solving of challenges experienced.
Planning	Planning for time between leaving DP and until next day due to return, with respect to meal plan compliance and management of ED behaviours. Using problem-solving and motivational interviewing approach.
Nutrition Group	Session delivered by dietitian. Providing information, education and skill development on relevant topics, e.g., starvation syndrome, regular and adequate eating, fluids, macronutrients, food rules, feared foods, metabolism, cooking, meal preparation, evaluating nutritional advice, and social eating.
Physical Recovery	Collaborative weighing, review of meal plan adherence and risk assessment (carried out individually).
Goal Review	Reviewing achievement of individual goals set over previous week and problem-solving.
Goal Setting	Setting 5 specific goals for week ahead to address ED behaviours, including setting a challenge snack to eat within DP supported meal and a goal to get back into normal life.
Flexible Thinking	Psychology-based group covering such topics as body image, perfectionism, self-compassion and also cognitive remediation therapy.
Coping Skills	Delivering dialectical behavioural therapy skills modules of mindfulness, distress tolerance, emotion regulation and interpersonal effectiveness.
Life and Relationships	Issues that impact life and relationships in people with an ED, e.g., sleep, assertive communication skills, motivation for recovering, social media literacy, reviewing values, vocational choices.
Mind	Cognitive-behaviour therapy (CBT) strategies for EDs, e.g., developing an understanding of how the ED is maintained, identifying and addressing unhelpful thinking styles, developing understanding of thought, feelings and behaviour connections.
Distress Tolerance	Independent practice of strategies to manage distress with clinician available to provide support and engagement. For example, playing games, practising mindfulness, distraction activities, art.
Mindfulness	Education on mindfulness and practising various mindfulness activities.
Creative Writing	Delivered by professional writer, ‘writer in residence’. Guiding patients through various creative writing tasks, including poetry.
Sensory	Developing skills and knowledge to use sensory approaches in developing regulation skills (e.g., self-soothing or alerting/arousal using the 5 senses).
Group Processes	Review group dynamics and norms, to identify and address any issues.

**Table 3:** Overview of day program formats.

Program Descriptors	Format 1	Format 2	Format 3
Date	August 2018 to August 2019	September 2019 to September 2020	October 2020 to July 2021
Admission lengths	Determined on an individual case-by-case basis, typically during admission		Pre-determined typically prior to commencement
Meal support	Morning tea at start of admission.		
	Lunch and afternoon tea on each DP day		
Dinner meal support	3 per week	1 per week	
		1 take home meal per week	
Crisis support	Provided on an individual basis		Limiting provision of crisis support. Participants required to cease and resume once manageable and able to re-engage.
Patient relationships	Not encouraged, no formal requirements		Not permitting socialising with other patients outside of DP.
Total DP hours	25.25	22.25	22.25

**Table 4:** Descriptors and comparisons between the three groups.

Variable	Group 1	Group 2	Group 3	ANOVA Comparison
	Mean (SD)	Mean (SD)	Mean (SD)	*F, p*
	*n* = 28	*n* = 17	*n* = 11	
Age	25.78 (8.65)	26.74 (10.41)	21.70 (5.23)	1.20, 0.31
Duration of admission (weeks)	9.79 (5.95)	12.53 (5.48)	10.40 (4.17)	1.33, 0.28
BMI: underweight	17.64 (0.79)	15.15 (5.42)	16.57 (1.18)	1.18, 0.33
Motivated to recover	73.96 (17.96)	76.25 (21.35)	79.00 (20.41)	0.27, 0.76
Ready to change	63.19 (26.23)	56.81 (29.11)	57.70 (30.68)	0.31, 0.73
Confidence	55.33 (24.43)	70.56 (27.05)	62.82 (22.59)	1.90, 0.16
	*N* (%)	*N* (%)	*N* (%)	*X*^2^ (df), *p*
BMI < 18.5	9 (50)	10 (75)	6 (55)	4.60 (2), 0.10
Female	26 (93)	17 (100)	11 (100)	2.07 (2), 0.36
Anorexia Nervosa	13 (46)	14 (82)	8 (73)	8.63 (6), 0.20

**Table 5:** Within-group effect size change (corrected for correlations between admission and discharge) regardless of format (*n* = 56).

Variable	Admission	Discharge	Cohen’s *d* Effect Size
	Mean (SE)	Mean (SE)	(95% Confidence Intervals)
EDE-Q	4.23 (0.19)	2.82 (0.22)	−1.16 (−1.56: −0.76)
CIA	34.86 (1.48)	23.99 (1.80)	−0.99 (−1.39: −0.60)
DASS	74.35 (3.80)	58.00 (4.57)	−0.68 (−1.06: −0.30)
BMI < 18.5 (*n* = 24)	16.45 (0.73)	19.30 (0.37)	0.80 (0.21:1.39)
Weight (kg)	47.10 (1.03)	53.13 (1.11)	1.66 (1.56:2.87)

## Data Availability

The data that support the findings of this study are available from the corresponding author upon reasonable request.
